# Sound Source Localization Using a Convolutional Neural Network and Regression Model

**DOI:** 10.3390/s21238031

**Published:** 2021-12-01

**Authors:** Tan-Hsu Tan, Yu-Tang Lin, Yang-Lang Chang, Mohammad Alkhaleefah

**Affiliations:** Department of Electrical Engineering, National Taipei University of Technology, Taipei 10608, Taiwan; thtan@ntut.edu.tw (T.-H.T.); ttommy472tw@gmail.com (Y.-T.L.); ylchang@ntut.edu.tw (Y.-L.C.)

**Keywords:** deep learning, sound source localization, convolutional neural network, regression model

## Abstract

In this research, a novel sound source localization model is introduced that integrates a convolutional neural network with a regression model (CNN-R) to estimate the sound source angle and distance based on the acoustic characteristics of the interaural phase difference (IPD). The IPD features of the sound signal are firstly extracted from time-frequency domain by short-time Fourier transform (STFT). Then, the IPD features map is fed to the CNN-R model as an image for sound source localization. The Pyroomacoustics platform and the multichannel impulse response database (MIRD) are used to generate both simulated and real room impulse response (RIR) datasets. The experimental results show that an average accuracy of 98.96% and 98.31% are achieved by the proposed CNN-R for angle and distance estimations in the simulation scenario at SNR = 30 dB and RT60 = 0.16 s, respectively. Moreover, in the real environment, the average accuracies of the angle and distance estimations are 99.85% and 99.38% at SNR = 30 dB and RT60 = 0.16 s, respectively. The performance obtained in both scenarios is superior to that of existing models, indicating the potential of the proposed CNN-R model for real-life applications.

## 1. Introduction

Localization technologies are widely used in everyday applications, such as navigation, human–computer interaction, surveillance, rescue, and smart monitoring [1,2]. Global positioning system (GPS) is the most frequently used technology for outdoor positioning [3,4]. However, GPS accuracy is degraded when it is used in indoor environments due to obstacles blocking the signal’s propagation [5,6]. Consequently, a number of technologies, such as infrared (IR), Bluetooth, and Wi-Fi, have been developed to address the challenge of indoor positioning. These technologies have become widely used for indoor localization and positioning in recent years [7]. The propagation path of radio signals can be line-of-sight (LOS) or non-line-of-sight (NLOS) in indoor environments [8]. However, the signals of indoor positioning technologies must be propagated in LOS conditions in order to produce accurate location estimates [9]. Although IR offers high localization accuracy, its signal can be easily obscured by obstacles [10]. Bluetooth and Wi-Fi have the advantage of strong penetrating power, which can penetrate through indoor obstacles [11,12]. Nevertheless, Bluetooth is disadvantaged by its short range, and Wi-Fi requires high costs of hardware installation and maintenance [13]. Sound has the advantages of strong penetrating power, simple construction, and low cost [14]. Additionally, sound includes a tone, timbre, and other features, which make it more effective than other technologies [15]. For example, the frequency of sound emitted from different locations can be distinguished efficiently, and multiple sound sources can be located at the same time. Therefore, sound source localization (SSL) has attracted much attention in recent years [16,17,18].

Currently, two types of sound source localization methods are generally used in the literature. First, the microphone array methods use the microphone array as a receiving end to determine the direction of the sound source. The microphone arrays can be divided into linear arrays, circular arrays, and distributed arrays. Second, human ear analysis methods identify the sound source via simulating the signal received by the human ear. It was shown in [19,20,21,22,23,24] that binaural beamforming-based methods can achieve high noise reduction and sound sources preservation and localization. Microphone array-based methods can be further divided into four approaches under different acoustic characteristics as follows [25,26,27,28]:

1. Beamforming: calculate the input signal power, phase, and amplitude of each receiving point through beamforming technology, and calculate the azimuth angle of the sound source with the greatest probability.
2. Time difference of arrival (TDOA): the time difference between the signals’ arrival at two or more receiving points is combined with the spatial information of these receiving points to infer the azimuth of the sound source [29].
3. High-resolution spectrum estimation (HRSE): the signal at the receiving point is used to calculate the correlation between the spatial and spectral characteristics to obtain the azimuth angle of the sound source [30].
4. Neural network (NN): train a NN model using a large amount of data to find audio patterns for multiple acoustic sources localization [31].

Recently, various deep neural networks (DNNs) were employed for sound source localization. Chakrabarty et al. [32] proposed a CNN-based supervised learning (CNN-SL) approach to estimate the direction of arrival (DOA) of multiple speakers. The phase component of the STFT coefficients of the received microphone signals are directly fed into the CNN, and the features for DOA estimates are learned during the training process. The ability of the DOA estimation method to accurately adapt to unseen acoustic conditions is pretty robust. However, this method is highly dependent on the time-varying source signal [33]. Yiwere et al. [34] presented a sound source distance estimation (SSDE) approach by using a convolutional recurrent neural network (CRNN). The CRNN is trained using log-scaled mel spectrograms extracted from single-channel audio signals as input features. The transformation of the audio signals to images allows the convolutional layers of the network to extract distance-dependent features from the audio signals. The experimental results showed that the CRNN model can achieve a high level of accuracy. Another interesting research work [35] proposed an indoor sound source regional localization method based on a convolutional neural network (CNN). The sound source signal is converted into a spectral map and fed into the CNN for regional localization. The simulation results showed that the CNN can bring better robustness and generalization with different SNRs. Pang et al. [36] introduced a binaural sound source localization (SSL) method based on time–frequency CNN (TF-CNN) with multitask learning to simultaneously localize azimuth and elevation under various acoustic conditions. The IPD and interaural level difference (ILD) are first extracted from the received binaural signals, then each or both of them are fed to the SSL neural network. The experimental results illustrated that the proposed method can achieve comparable localization performance. Nevertheless, such methods are restricted to certain ranges or areas.

This research aims to construct an indoor localization model based on the characteristics of the sound spectrum, which can estimate the azimuth angle and distance of the indoor speaker. The CNN is used to automatically extract features and increase their versatility and robustness by training the model to resist noise. Previous works used classification functions to normalize the output of a CNN to a probability distribution over the output target. However, the output of classification functions is a discrete value, and hence, it does not predict the exact value in the case of continuous variables. Unlike the previous studies, our CNN uses a regression function instead of a classification function because it is better suited for the continuous variable output. Additionally, this research uses the Pyroomacoustics [37] platform to quickly construct a virtual three-dimensional space and generate a room impulse response (RIR) with spatial sound signals. Moreover, real space signals are synthesized with a multi-channel impulse response database [38]. In addition, the signal dataset is converted into a time-frequency domain signal through STFT and then is converted to an IPD feature map to be fed to the CNN model. Finally, the distribution of output values with the regression model are observed to find the best configuration of the model through training and evaluate the performance of the model in different environments.

## 2. Proposed Methods

**Figure 1:**
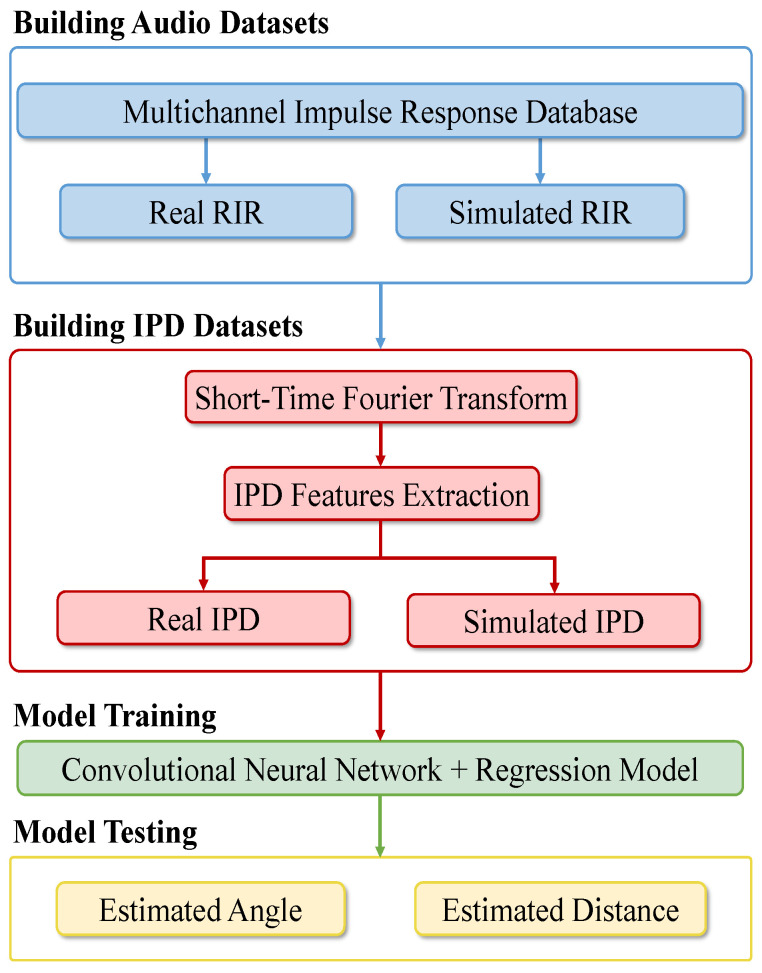
Flow chart of the proposed system.

The overall flow chart of the proposed sound source localization system is demonstrated in Figure 1. The sound database signal is firstly convolved with the real and simulated RIR to obtain a new signal with spatial effect. Then the STFT of the new signal is obtained, and the IPD features are extracted. Finally, the CNN-R model is trained on the IPD features to estimate the angle and distance of the sound source. Notably, the IPD image sets are divided into 70% training set, 10% validation set, and 20% test set.

### 2.1. Data Collection

This research uses the CMU_ARCTIC database, which is a speech database in CMU_ARCTIC speech synthesis databases [39], established by the Language Technologies Institute of Carnegie Mellon University, USA. This database is mainly used in the research of speech synthesis. The content of the corpus database was selected by the non-copyright center of Project Gutenberg, which is about 1150 sentences. An audio of two males and two females with American English accents were collected. The recording format is 16 bits, the sampling rate is 32 KHz, and the length of each sentence is 3 seconds. The database has a total of 4528 audio files.

#### 2.1.1. Simulated Room Database

**Figure 2:**
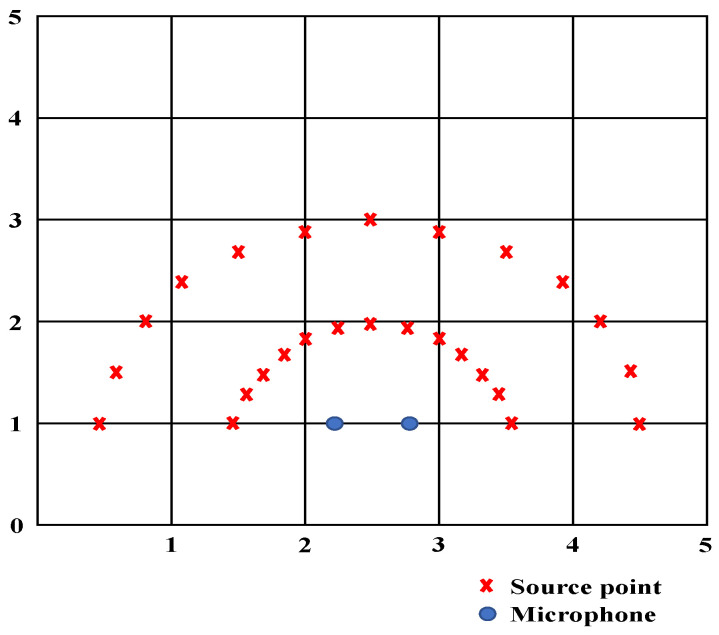
Simulated space configuration diagram.

In this research, we built our own simulated spatial dataset, and the RIR was simulated by using the Pyroomacoustics Python platform [37]. The length and the width of the generated space are 5 × 5, 5 × 6, 6 × 6, 7 × 6, and 7 × 7 (m${}^{2}$), and the height is 2.5 m. The position of the two microphones is (x = (width/2) ± 0.3, y = 1, z = 1). Figure 2 shows an example of a 5 × 5 space. The microphones are located at (2.2, 1, 1) and (2.8, 1, 1). The sound source point is 1 and 2 m from the midpoint of the two microphones. The angle is distributed from 0° to 180°, where every 15° is a step distance. In total, there are 26 source points. The sound database adopts the CMU_ARCTIC database. A total of 100 audio files are taken from the corpus of 4 participants. Convolution operations are performed at each sampling point to generate spatial sound effects, and we adjust RT60 and SNR to achieve data diversity.

#### 2.1.2. Real Room Database

**Figure 3:**
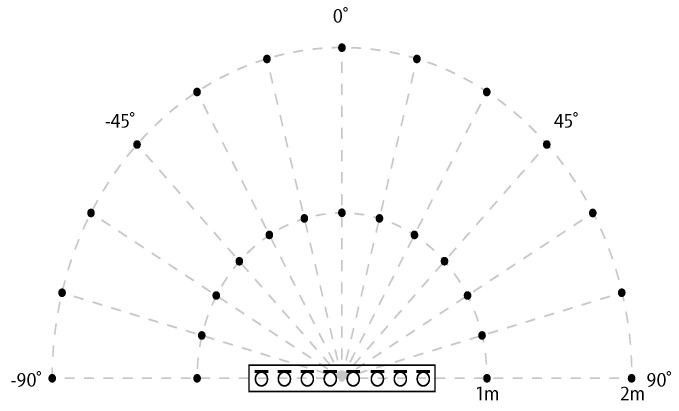
Multichannel Impulse Response Database measurement space configuration diagram [38].

This research uses the real room response database generated by the Multichannel Impulse Response Database. The database was constructed by the Institute of Communication Systems and Data Processing at RWTH Aachen University, Germany. These data can produce a variety of reverberation levels by changing the spatial scene. The database mainly has three different reverberation levels with reverberation times (RT60) of 0.16 s, 0.36 s, and 0.61 s, respectively [38]. On different grids in space, in the angular range of 90° to −90°, each 15° step is taken as a measurement point, and each measurement point is 1 m or 2 m away from the microphone array. There are 26 measurement points in total, as shown in Figure 3.

### 2.2. Data Processing

After the simulation and real space datasets are generated, data processing is still needed before the data can be fed to the model for training. First, the sound signal is transformed to the time-frequency domain signals through STFT, then an IPD feature map is produced by using the Hann window as a sliding window for STFT. The Hann window size is 2048 sampling points with 512 overlapping sampling points and a sampling rate of 14,400 Hz. IPD is a feature formed by converting two audio signals into frequency spectra and subtracting each phase. The intensity of the left and right phases can be observed. Unlike using two spectra for training, one IPD makes training faster. Hann’s window is used because it shows superior performance in random signals. The conversion formula is shown in Formula (1):

(1) $$Y_{m}\left(\omega ,k\right)=A_{m}\left(\omega ,k\right)e^{⋀(j\varphi_{m}\left(\omega ,k\right))},m=l,r$$

where $A_{m}$(ω,k) and $\varphi_{m}$(ω,k) are the components of amplitude and phase at frequency ω and time *k*, respectively. *l* and *r* are the left and right channels, respectively.

**Figure 4:**
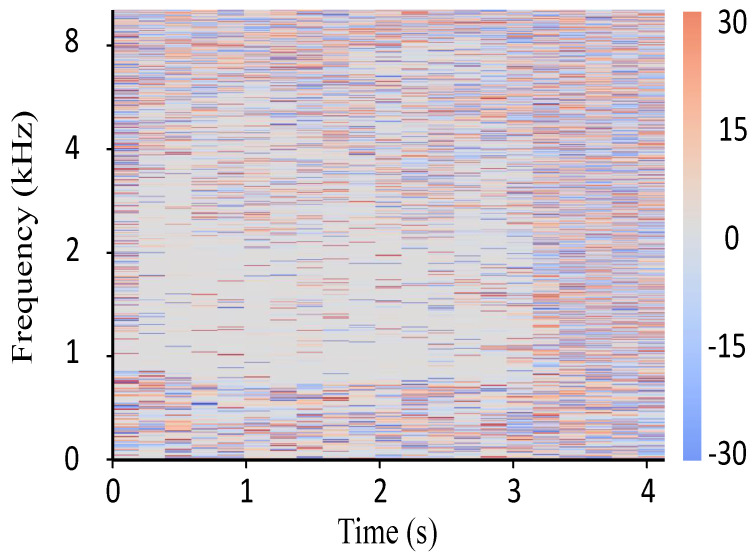
An example of the actual output of IPD where the color map represents the angle (°).

The IPD features are obtained based on the phase difference between the two receiving ends, and its formula can be expressed as follows:

(2) $$\varphi \left(\omega ,k\right)=∠\frac{Y_{l}\left(\omega ,k\right)}{Y_{r}\left(\omega ,k\right)}$$

where $Y_{l}$(ω,k) and $Y_{r}$(ω,k) are the left and right receiving signals. The IPD can be obtained by subtracting its phase components. In other words, the IPD is computed as the difference of the phase angles, and phase unwrapping is used on the phase image. Figure 4 is an example of the actual output of the IPD.

The dataset of the simulated IPD includes 400 audio records, 5 spatial sizes (5 × 5, 5 × 6, 6 × 6, 7 × 6, and 7 × 7 m${}^{2}$), 26 sampling points, 5 SNR (0, 5, 10, 20, and 30 dB), 3 RT60 (0.16, 0.36, and 0.61 s), and a total of 780,000 images. The dataset of Real IPD includes: 400 audio records, 1 spatial size (6 × 6 m${}^{2}$), 26 sampling points, 5 SNR (0, 5, 10, 20, and 30 dB), 3 RT60 (0.16, 0.36, and 0.61 s), and a total of 156,000 images. The nature of the noise is independent Gaussian white noise added to each channel, and it is computed as follows:

(3) $$SNR=10\;log_{10}\left(\frac{SignalPower}{NoisePower}\right)dB$$

### 2.3. The Proposed CNN-R Architecture

**Figure 5:**
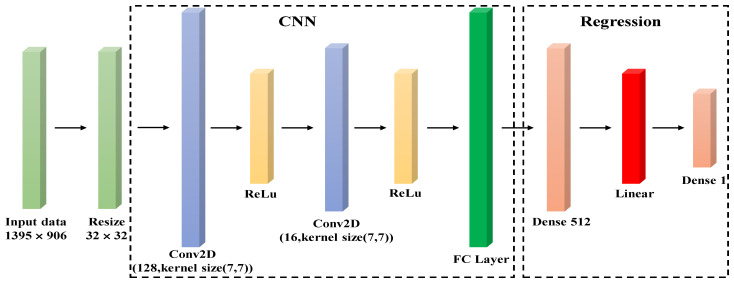
The architecture of CNN-R.

The proposed model is composed of two architectures, including CNN and regression architecture. First, the resolution of the original data is reduced from 1395 × 906 to 32 × 32 before training. The CNN is constructed by two convolutional layers. The first layer has 128 kernels and the size of the kernel is 77, the padding is same, and the stride is 1. The second convolutional layer has 16 kernels of the size, 7 × 7, the padding is the same, and the stride is 1. The ReLu activation function is used after each convolutional layer. The feature map output by the last layer of CNN is flattened by the fully connected (FC) layer and then is used to train the model. The regression model has three layers in total. The number of kernels in the first and second layers is 512, and the number of kernels in the third layer is set to 1 to generate the final output. The activation function used between each layer is linear. Figure 5 shows the overall architecture of the proposed CNN-R model used in this research. Additionally, Table 1 shows the experimental training setting.

The proposed CNN-R architecture is kept as small as possible to avoid overfitting. The simpler architecture is less likely to overfit [40]. The choice and numbers of layers were decided by trial and error. The main criteria were accuracy and MAE. When the accuracy and MAE are not improving on the validation dataset during the training stage, the training process is interrupted after a certain number of epochs, and then the structure of the model is modified. This process is repeated until the new structure produces satisfactory results. In the CNN part, the model started with one convolutional layer and one ReLu layer; however, the results were not satisfactory. Hence, we added one more convolutional layer followed by the ReLu layer, and the results were impressive. Additionally, the regression part, including Dense and the linear activation function, were used because the output of the FC layer is a 1D continuous variable output. Finally, a Dense layer was used to produce the value of angle and distance.

## 3. Experimental Results

The generated IPD dataset was divided into three parts: training, validation, and testing. We used the validation dataset to monitor the performance of our model during the training and adjust the model hyperparameters to find the optimal configuration. All the experiments were performed using a PC with Intel Core i7-7700, CPU 3.6 GHz, and 32 GB of RAM. The GPU was NVIDIA GeForce RTX 2070 with 8 GB of memory. The model was implemented in Python with TensorFlow.

### 3.1. Performance Evaluation Metrics

In this study, the regression model is used to evaluate the overall performance by comparing the difference between the predicted value and the actual value. According to the distribution of output values, the precision and accuracy (Acc.) are interpreted as follows:

1. High precision and high accuracy: the distribution of predicted values is concentrated and close to the target value.
2. High precision and low accuracy: the distribution of the predicted values is concentrated, but far from the target value.
3. Low precision and high accuracy: the distribution of the predicted values is more scattered, but around the target value.
4. Low precision and low accuracy: the distribution of the predicted values is more scattered and far from the target value.

**Figure 6:**
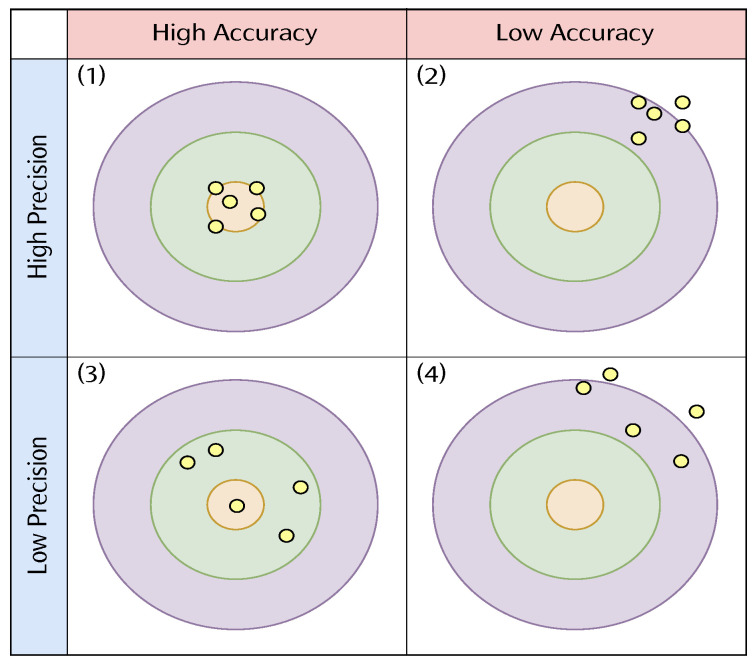
Performance evaluation criteria where yellow dots are the predicted values. (1) is high accuracy and high precision, which is the best scenario. (2) is low accuracy and high precision. (3) is high accuracy and low precision. (4) is low accuracy and low precision, which is the worst scenario.

Figure 6 illustrates the relationship between precision and accuracy with respect to the distribution of the predicted values. In this research, the mean absolute error (MAE) is used to measure the accuracy. MAE calculates the difference between the predicted value and the actual value. The formula is shown in (4):

(4) $$MAE=\frac{1}{N}\sum_{i=1}^{N}\left|y_{i}-\hat{y_{i}}\right|$$

Because the output of the regression model is a continuous value, this research uses the formula in (5) to evaluate the accuracy of the proposed CNN-R:

(5) $$Accuracy=\frac{N_{fine}}{N_{T}}$$

where $N_{T}$ is the total times of experiments, $N_{fine}$ is the number of the error which is less than the unit scale divided by 2. In this research, the angle estimation unit scale is 15° and the distance unit scale is 1 meter; therefore, the angle is 7.5° (15/2) and the distance estimation is 0.5 meter (1/2) as the baseline value. When the predicted value is less than the baseline value, it is considered to be a correct value. In this research, the experiments are mainly divided into two parts as follows:

1. Experiment 1 in a simulated environment consists of two parts: (i) a single acoustic environment is used to train the model for angle and distance estimation, and (ii) a multiple acoustic environment is used to train the model for angle and distance estimation.
2. Experiment 2 uses a real spatial sound dataset to train the model for angle and distance estimation.

### 3.2. Experiment 1

#### 3.2.1. Model Performance in a Single Acoustic Environment

In the experiment, the goal is to show the ability of the proposed CNN-R architecture to make correct predictions in different room dimensions under the same RT60. The use of different room dimensions is to avoid data overfitting and validate the performance of CNN-R. Additionally, the same RT60 is used to avoid environmental parameter changes. The experimental results show that the proposed CNN-R can be generalized and used in multiple acoustic environments. Table 2 shows the single acoustic environment configuration. The training set room includes 5 × 5, 6 × 5, 6 × 7, and 7 × 7 (m${}^{2}$). The SNRs are 0 dB and 5 dB, respectively. The RT60 is 0.16 s. On the other hand, the testing set room is 6 × 6 (m${}^{2}$). The SNRs are 10 dB, 20 dB, and 30 dB, respectively. The RT60 is 0.16 s.

Table 3 and Table 4 show the model performance for angle and distance estimation in the single acoustic environment under three SNR scenarios, respectively.

**Figure 7:**
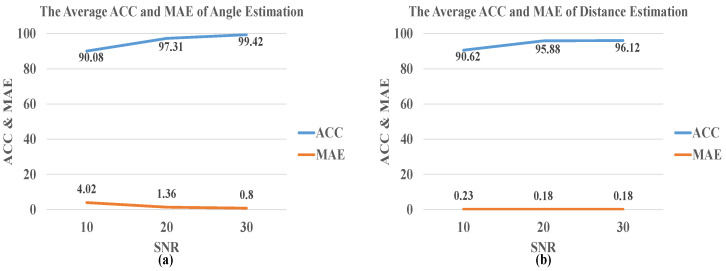
The average accuracy and MAE of (**a**) angle and (**b**) distance estimation by CNN-R in a single acoustic environment, where SNR = 10 dB, 20 dB, 30 dB, and RT60 = 0.16 s.

Figure 7 shows the average of accuracy and MAE for the angle and distance estimation in the single acoustic environment under SNR = 10 dB, 20 dB, 30 dB, and RT60 = 0.16 s. In the single acoustic environment, the accuracy of the angle and distance estimation increases as the SNR increases. When the SNR is greater than 20 dB, the angle and distance accuracy can reach 99.42% and 96.12%, respectively. Additionally, the MAE is reduced to 0.8° and 0.18 m, and the RMSE is reduced to 1.32° and 0.14 m. The accuracy of the angle estimation model in each SNR is better than the distance estimation model.

#### 3.2.2. Model Performance in a Multiple Acoustic Environment

Table 5 shows the multiple acoustic environment configuration. The training set room includes 5 × 5, 6 × 5, 6 × 7, and 7 × 7 (m${}^{2}$). The SNRs are 0 dB, 5 dB, and 10 dB, respectively. The RT60 are set to 0.16 s, 0.36 s, and 0.61 s, respectively. In order to be different from the training environment, the testing set room is 6 × 6 (m${}^{2}$). The SNRs are set to 10 dB, 20 dB, and 30 dB, respectively. The RT60 are 0.16 s, 0.36 s, and 0.61 s, respectively, which are the same as the training set.

Table 6 shows the model performance for angle estimation in the multiple acoustic environment under SNR = 10, 20, 30 dB, and three RT60 scenarios. Table 7 shows the model performance for distance estimation in the multiple acoustic environment under SNR = 10, 20, and 30 dB, and three RT60 scenarios.

**Figure 8:**
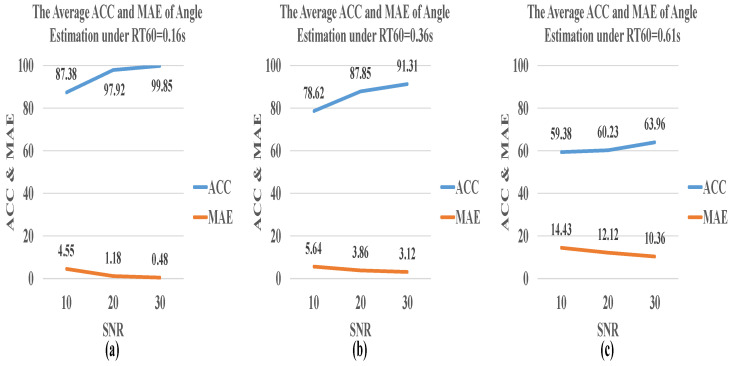
The average ACC and MAE of angle estimation by CNN-R in a multiple acoustic environment where SNR = 10 dB, 20 dB, and 30 dB, and (**a**) RT60 = 0.16 s, (**b**) RT60 = 0.36 s, and (**c**) RT60 = 0.61 s.

**Figure 9:**
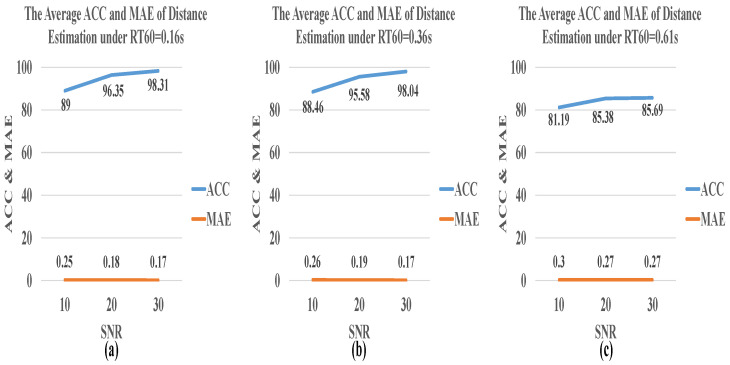
The average ACC and MAE of distance estimation by CNN-R in a multiple acoustic environment where SNR = 10 dB, 20 dB, and 30 dB, and (**a**) RT60 = 0.16 s, (**b**) RT60 = 0.36 s, and (**c**) RT60 = 0.61 s.

Figure 8 and Figure 9 show the model performance for the angle and distance estimation in the multiple acoustic environments under SNR = 10 dB, 20 dB, 30 dB, and RT60 = 0.16 s, 0.36 s, 0.61 s, respectively. In the multiple acoustic environment, two acoustic spaces, RT60 = 0.36 s and RT60 = 0.61 s, were added. The overall accuracy increases with the increase of SNR and the decrease of RT60. When RT60 = 0.61 s, the performance of angle estimation is not satisfactory, and the average accuracy is 61.19%. However, if RT60 is reduced to 0.36 s, the accuracy can be greatly increased by about 20%. Moreover, MAE and RMSE drop sharply at RT60 = 0.16 s. Nevertheless, the best performance for angle estimation is achieved when SNR = 30 dB, MAE = 1.96, and RMSE = 1.64, where the accuracy is 98.96%.

### 3.3. Experiment 2

#### The Model Performance in a Real Acoustic Environment

Table 8 shows the real acoustic environment configuration. The training set room is 6 × 6 (m${}^{2}$). SNR are 0 dB, 5 dB, and 10 dB. The RT60 are 0.16 s, 0.36 s, and 0.61 s. The test set room is 6 × 6 (m${}^{2}$). The SNR are 10 dB, 20 dB, and 30 dB. The RT60 are 0.16 s, 0.36 s, and 0.61 s, which is similar to the training set.

Table 9 shows the model performance for distance estimation in the real acoustic environment under SNR = 10, 20, and 30 dB, and RT60 = 0.16 s, 0.36 s, and 0.61 s, respectively.

Table 10 shows the model performance for angle estimation in the real acoustic environment under SNR = 10, 20, and 30 dB, and RT60 = 0.16 s, 0.36 s, and 0.61 s, respectively.

Table 11 shows the average Acc and MAE of the proposed model for angle and distance estimation in real acoustic environments, where SNR = 10 dB, 20 dB, and 30 dB, and RT60 = 0.16 s, 0.36 s, and 0.61 s, respectively. In a real acoustic environment, the angle estimation accuracy increases and the error decreases as SNR increases and RT60 decreases. Moreover, when the SNR is greater than 20 dB, the accuracy obtained is higher than 96%, and the MAE is less than 1.7°. The accuracy of distance estimation is also improved with the increase in SNR. Overall, the accuracy is higher than 95% when SNR = 20 dB and 30 dB. The Acc. and MAE of each RT60 are stable when SNR is greater than 20 dB. Table 12 shows the accuracy of CNN-R for angle and distance estimation compared to other methods based on the multi-channel impulse response database [38].

**Figure 10:**
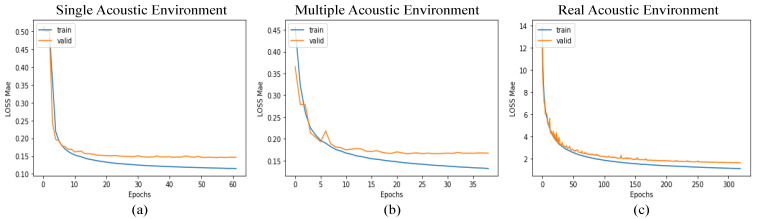
The training–validation loss curves of CNN-R, where (**a**) is the performance of CNN-R in a single acoustic environment. (**b**) is the performance of CNN-R in a multiple acoustic environment. (**c**) is the performance of CNN-R in a real acoustic environment.

The training–validation loss curves for the proposed CNN-R in a single acoustic environment, multiple acoustic environment, and real acoustic environment are shown in Figure 10. Unlike the single acoustic environment and multiple acoustic environment, the loss in real acoustic environment gradually reduces and slowly converges as the number of epochs increases. Moreover, note in Figure 10c that the training loss curve and validation loss curve behave similarly, which implies that the proposed CNN-R model can be generalized and does not suffer from overfitting.

## 4. Discussion

This research aims to establish a general sound localization model. The results of a single acoustic environment in Experiment 1 show that under different room conditions, the test model can still effectively estimate the angle and distance in a single acoustic environment, and it will be more accurate as the SNR increases. In the multiple acoustic environments, good estimation performance can also be obtained under different room conditions. When RT60 = 0.61 s, the accuracy is relatively insufficient. However, as the SNR increases, the accuracy can be effectively improved. The model proposed in this research has the best performance in the simulated room where the RT60 is less than 0.36 s and the SNR is greater than 20 dB. In addition, in the real acoustic environment of Experiment 2, the overall accuracy is enhanced significantly, verifying the practicability of our proposed model in a real acoustic environment. The experimental results show that the MAE of the model for the angle estimation is smaller than the distance estimation, which means that the error between the predicted value and the actual value is small. Nonetheless, the RMSE of the model for angle estimation is greater than the distance estimation, which means that a small number of predicted values has large variations; hence, the model for angle estimation has high accuracy. However, the precision is low. On the other hand, the model performance for distance estimation has high accuracy and high precision.

Comparing the results of the proposed CNN-R in the multiple acoustic environment in Experiment 1 with the results in the real acoustic environment in Experiment 2, it can clearly be seen that under the same environmental acoustic parameters, the accuracy of the model trained in the real environment is higher than that of the simulated acoustic environment. The reason for this result is that when generating simulated room sound effects, the only parameters we can adjust are SNR and RT60. However, in the real environment, the parameters that affect sound propagation are more complex. Therefore, the model trained with the simulation dataset has insufficient features, which affects the learning of the model, resulting in a decrease in accuracy. The experimental results show that the accuracy of the distance estimation is better than that of the angle estimation. The reason is that there are 13 target values for the angle estimation and only 2 target values for the distance estimation, which increases the complexity of the angle estimation model weight training and makes the weight distribution uneven.

Taking Table 3 and Table 4 as an example, when SNR = 10 dB, the accuracy of the angle estimation is between 71% and 100%. The accuracy close to 90° is higher, and the accuracy close to 0° or 180° decreases on both sides. The accuracy of the distance estimation is distributed between 87.08% and 94.15%, and the distribution of the accuracy of the distance estimation is more concentrated than that of the angle estimation. Moreover, the Acc. is low and MAE is high due to the small number of training samples in the single acoustic environment compared to the multiple acoustic environment. Additionally, in general, the accuracy drops significantly when the value of RT60 increases, except when the angle is 90 degrees in the multiple acoustic environment. One limitation of the proposed model might be the offline design. Future work will focus on improving the proposed model for real-time positioning. Additionally, the proposed model still needs further enhancement for multiple sound source localization.

## 5. Conclusions and Future Works

In this paper, an original sound source localization model was developed by combining a convolutional neural network and a regression model (CNN-R). Simulated and real sound datasets were generated to perform the experiments. Initially, the sound signals were transformed into time-frequency signals through STFT, and then IPD feature maps were calculated from the time-frequency signals. These were then fed into the CNN-R model for a series of experiments. The evaluation metrics of Acc., MAE, and RMSE were used to evaluate the performance of the proposed model. The experimental results in the simulated acoustic scenarios showed that the proposed model can effectively estimate the angles and distances in a single or multiple acoustic environments under different spatial conditions. When SNR is greater than 10 dB and RT60 is less than 0.61s, the accuracy of the angle and distance estimations can reach, on average, more than 95%. Additionally, when SNR = 30 dB and RT60 = 0.16 s, the accuracies of the angle and distance estimations can reach 98.96% and 98.31%, respectively. On the other hand, the experimental results in the real acoustic scenarios showed that when the SNR is greater than 20 dB, the accuracy of the angle and distance estimation exceeds 96%. Furthermore, when SNR = 30 dB and RT60 = 0.16 s, the accuracies of the angle and distance estimations reach 99.85% and 99.38%, respectively. In comparison to the existing methods, the experimental results also showed that the proposed CNN-R outperforms the existing methods in terms of the angle and distance estimation accuracies. Future work will study the combination of other acoustic features, such as ILD, to make the features richer. Moreover, the impact of more acoustic environments on the accuracy will also be investigated.

## Figures and Tables

**Table 1:** The settings of the training option used in the single acoustic environment.

Hyperparameters	Configurations
Optimizer	Adam
Loss Function	MAE
Learning Rate	0.001
Decay	$10^{-3}$
Execution Environment	GPU
Batch Size	64

**Table 2:** A single acoustic environment configuration.

	Training Set	Test Set
Room size (m^2^)	5 × 5, 6 × 5, 6 × 7, 7 × 7	6 × 6
SNR (dB)	0, 5	10, 20, 30
RT60 (s)	0.16	0.16

**Table 3:** Performance of angle estimation by CNN-R in a single acoustic environment.

	SNR = 10 dB		SNR = 20 dB		SNR = 30 dB	
Angle (°)	Acc. (%)	MAE (°)	Acc. (%)	MAE (°)	Acc. (%)	MAE (°)
0	71.00	15.67	90.50	4.28	99.00	1.68
15	87.50	3.13	98.00	1.43	100.00	0.91
30	83.50	5.01	94.00	2.08	97.00	1.66
45	88.50	4.32	97.00	1.60	100.00	0.67
60	99.00	1.02	100.00	0.54	100.00	0.57
75	100.00	0.53	100.00	0.32	100.00	0.34
90	100.00	0.47	100.00	0.22	100.00	0.18
105	99.50	0.69	100.00	0.43	100.00	0.53
120	100.00	0.72	100.00	0.40	100.00	0.40
135	85.50	5.27	97.50	1.49	99.50	0.52
150	82.00	4.59	91.50	2.46	97.50	1.62
165	94.50	2.95	99.50	0.86	100.00	0.56
180	80.00	7.85	97.00	1.53	99.50	0.81
Average	90.08	4.02	97.31	1.36	99.42	0.8

**Table 4:** Performance of distance estimation by CNN-R in a single acoustic environment.

	SNR = 10 dB		SNR = 20 dB		SNR = 30 dB	
Distance (m)	Acc. (%)	MAE (m)	Acc. (%)	MAE (m)	Acc. (%)	MAE (m)
1	87.08	0.25	95.69	0.18	96.92	0.17
2	94.15	0.21	96.08	0.18	95.31	0.19
Average	90.62	0.23	95.88	0.18	96.12	0.18

**Table 5:** A multiple acoustic environment configuration.

	Training Set	Testing Set
Room size (m${}^{2}$)	5 × 5, 6 × 5, 6 × 7, 7 × 7	6 × 6
SNR (dB)	0, 5, 10	10, 20, 30
RT60 (s)	0.16, 0.36, 0.61	0.16, 0.36, 0.61

**Table 6:** Performance of angle estimation by CNN-R in a multiple acoustic environment at SNRs of 10, 20, and 30 dB, respectively.

		RT60 = 0.16 s		RT60 = 0.36 s		RT60 = 0.61 s	
Angle (°)	SNR	Acc. (%)	MAE (°)	Acc. (%)	MAE (°)	Acc. (%)	MAE (°)
0	10	80.50	05.32	85.00	04.32	59.50	10.69
	20	93.00	02.66	97.50	02.69	81.50	05.57
	30	99.00	02.39	99.50	02.02	87.00	04.29
15	10	96.00	02.82	80.00	05.17	31.50	17.70
	20	97.50	02.67	82.50	04.38	35.50	18.60
	30	99.50	02.15	93.50	03.88	44.00	15.74
30	10	91.00	03.24	58.00	08.55	44.50	21.34
	20	96.50	02.47	61.00	08.31	48.50	24.91
	30	96.00	02.19	63.50	06.54	48.50	25.24
45	10	89.50	03.45	79.50	06.06	45.00	19.54
	20	96.50	02.16	93.50	03.16	58.50	14.38
	30	99.50	01.56	95.50	02.56	65.00	12.35
60	10	86.50	03.90	80.00	04.92	60.50	08.24
	20	98.00	02.17	94.50	03.63	63.50	07.24
	30	100.0	01.56	97.00	03.43	60.50	06.92
75	10	95.50	03.10	80.59	05.05	71.00	05.89
	20	100.0	01.57	98.00	02.24	81.00	04.80
	30	100.0	01.16	96.50	02.00	81.00	04.74
90	10	100.0	00.85	99.50	00.95	100.0	00.78
	20	100.0	00.52	100.0	00.50	100.0	00.52
	30	100.0	00.51	100.0	00.44	100.0	00.41
105	10	98.50	01.66	84.50	04.14	58.00	09.04
	20	99.50	01.02	97.50	01.79	61.50	08.28
	30	100.0	01.06	99.50	01.59	71.50	06.19
120	10	87.00	03.73	76.50	07.40	43.00	14.05
	20	98.00	01.81	84.00	04.59	49.50	10.04
	30	99.50	01.37	95.50	02.56	51.50	08.14
135	10	83.50	03.80	64.00	09.93	29.00	32.05
	20	95.00	02.19	79.00	07.12	44.50	24.60
	30	99.50	01.38	81.00	04.95	49.50	19.69
150	10	93.50	02.97	88.50	03.45	58.00	11.81
	20	95.50	02.46	94.00	02.50	61.50	11.39
	30	99.50	01.99	97.00	02.07	65.00	10.48
165	10	98.00	02.62	77.00	05.04	60.50	13.61
	20	100.0	01.84	75.50	05.01	45.50	10.05
	30	100.0	01.79	81.00	04.76	51.50	07.10
180	10	72.00	06.42	69.00	08.39	33.50	22.87
	20	98.50	02.31	85.00	04.27	52.00	17.15
	30	94.00	02.85	87.50	03.80	56.50	13.37
Average	10	90.12	03.37	78.62	05.64	53.38	14.43
	20	97.54	01.99	87.85	03.86	60.23	12.12
	30	98.96	01.69	91.31	03.12	63.96	10.36

**Table 7:** Performance of distance estimation by CNN-R in a multiple acoustic environment at SNRs of 10, 20, and 30 dB, respectively.

		RT60 = 0.16 s		RT60 = 0.36 s		RT60 = 0.61 s	
Distance (m)	SNR	Acc. (%)	MAE	Acc. (%)	MAE	Acc. (%)	MAE
1	10	86.00	00.26	84.31	00.29	76.46	00.34
	20	96.77	00.17	94.77	00.20	85.08	00.27
	30	98.46	00.16	98.38	00.16	89.08	00.24
2	10	92.00	00.24	92.62	00.23	85.92	00.26
	20	95.92	00.19	96.38	00.18	85.69	00.26
	30	98.15	00.18	97.69	00.18	82.31	00.30
Average	10	89.00	00.25	88.46	00.26	81.19	00.30
	20	96.35	00.18	95.58	00.19	85.38	00.27
	30	98.31	00.17	98.04	00.17	85.69	00.27

**Table 8:** The real acoustic environment configuration.

	Training Set	Test Set
Room size (m${}^{2}$)	6 × 6	6 × 6
SNR (dB)	0, 5, 10	10, 20, 30
RT60 (s)	0.16, 0.36, 0.61	0.16, 0.36, 0.61

**Table 9:** Performance of distance estimation by CNN-R in a real acoustic environment at SNRs of 10, 20, and 30 dB, respectively.

		RT60 = 0.16 s		RT60 = 0.36 s		RT60 = 0.61 s	
Distance (m)	SNR	Acc. (%)	MAE (°)	Acc. (%)	MAE (°)	Acc. (%)	MAE (°)
1	10	88.08	00.25	95.31	00.20	94.62	00.21
	20	98.54	00.15	99.31	00.14	99.23	00.14
	30	99.92	00.14	99.92	00.13	99.85	00.13
2	10	90.23	00.25	89.46	00.24	92.23	00.23
	20	97.62	00.17	97.85	00.16	97.31	00.16
	30	98.85	00.15	99.69	00.14	99.15	00.15
Average	10	89.15	00.25	92.38	00.22	93.42	00.22
	20	98.08	00.16	98.58	00.15	98.27	00.15
	30	99.38	00.14	99.81	00.13	99.50	00.14

**Table 10:** Performance of angle estimation by CNN-R in a real acoustic environment at SNRs of 10, 20, and 30 dB, and RT60 = 0.16 s, 0.36 s, and 0.61 s.

		RT60 = 0.16 s		RT60 = 0.36 s		RT60 = 0.61 s	
Angle (°)	SNR	Acc. (%)	MAE (°)	Acc. (%)	MAE (°)	Acc. (%)	MAE (°)
0	10	88.00	04.05	91.50	04.03	79.00	10.15
	20	98.50	01.66	99.50	01.28	97.00	02.62
	30	100.0	00.79	99.50	00.77	99.50	01.53
15	10	80.00	04.62	89.00	05.08	92.50	03.40
	20	95.00	02.36	95.00	02.48	99.00	01.36
	30	99.00	01.29	99.50	01.08	99.50	00.97
30	10	95.00	02.35	81.00	10.36	77.50	12.64
	20	99.50	00.56	97.50	01.81	95.00	02.08
	30	100.0	00.37	100.0	00.44	99.00	00.55
45	10	87.00	04.49	91.50	03.08	80.50	07.85
	20	99.50	00.64	99.50	00.46	97.50	00.78
	30	100.0	00.32	100.0	00.27	100.0	00.30
60	10	86.00	05.87	86.00	06.12	83.00	07.33
	20	99.50	00.54	99.50	00.46	98.50	00.88
	30	100.0	00.28	100.0	00.29	100.0	00.27
75	10	100.0	01.46	78.50	10.97	82.50	07.85
	20	100.0	00.38	98.00	01.52	99.00	00.90
	30	100.0	00.32	96.50	00.40	100.0	00.35
90	10	88.00	04.29	85.00	05.03	72.50	12.51
	20	99.50	00.81	99.00	00.77	93.50	02.50
	30	100.0	00.38	100.0	00.40	99.00	00.54
105	10	86.00	05.98	78.50	11.36	61.50	16.93
	20	98.50	00.82	97.50	01.49	95.50	01.78
	30	100.0	00.28	100.0	00.26	100.0	00.32
120	10	93.00	02.74	86.00	04.12	78.00	10.82
	20	98.50	00.58	98.00	01.45	96.50	01.65
	30	100.0	00.21	100.0	00.23	100.0	00.29
135	10	85.50	05.80	87.50	05.43	82.50	06.41
	20	98.50	00.81	98.00	01.04	98.50	01.17
	30	100.0	00.26	100.0	00.40	100.0	00.48
150	10	82.50	08.74	91.00	02.90	78.50	09.86
	20	94.00	02.92	100.0	00.55	92.50	02.60
	30	99.00	00.55	100.0	00.29	99.50	00.38
165	10	92.00	02.87	91.00	03.02	77.50	09.77
	20	99.50	00.91	99.50	01.01	97.00	01.41
	30	100.0	00.53	99.50	00.68	100.0	00.59
180	10	76.50	05.84	81.50	05.99	82.00	09.24
	20	92.50	02.37	99.00	01.02	97.50	01.61
	30	100.0	00.72	100.0	00.45	100.0	01.12
Average	10	87.38	04.55	86.00	06.02	79.04	09.06
	20	97.92	01.18	98.46	01.18	96.69	01.64
	30	98.85	00.48	99.85	00.46	99.73	00.59

**Table 11:** The average Acc. and MAE of angle and distance estimation by CNN-R in a real acoustic environment where SNR = 10 dB, 20 dB, and 30 dB, and RT60 = 0.16 s, 0.36 s, and 0.61 s, respectively.

	Angle			Distance		
RT60 (s)	SNR (dB)	Acc. (%)	MAE (°)	SNR (dB)	Acc. (%)	MAE (m)
0.16	10	87.38	04.55	10	89.15	00.28
	20	97.92	01.18	20	98.08	00.16
	30	99.85	00.48	30	99.38	00.14
0.36	10	86.00	06.02	10	92.38	00.22
	20	98.46	01.18	20	98.58	00.15
	30	99.85	00.46	30	99.81	00.13
0.61	10	79.04	09.60	10	93.42	00.22
	20	96.69	01.64	20	98.27	00.15
	30	99.73	00.59	30	99.50	00.14

**Table 12:** Comparative results of angle and distance estimation based on the multi-channel impulse response database in a real acoustic environment at SNR = 30 dB and RT60 = 0.16 s.

Method	Average Angle (0–180°) Acc.	Average Distance (1–2 m) Acc.
CNN-SL [32]	90.25%	88.85%
CRNN [34]	87.37%	85.64%
CNN [35]	98.51%	98.09%
TF-CNN [36]	95.18%	94.66%
CNN-R	99.85%	99.38%
